# Trends of ED visits, admissions, and deaths for pediatric traumatic brain injury comparing sport and non-sport mechanisms

**DOI:** 10.1186/s40621-019-0207-x

**Published:** 2019-05-29

**Authors:** Holly R. Hanson, Michael A. Gittelman, Wendy J. Pomerantz

**Affiliations:** 1 Division of Pediatric Emergency Medicine, Department of Pediatrics, Monroe Carell Jr. Children’s Hospital at Vanderbilt, 2200 Children’s Way, Nashville, TN 37232 USA; 2 Division of Emergency Medicine, Department of Pediatrics, Cincinnati Children’s Hospital Medical Center, University of Cincinnati, 3333 Burnet Avenue, MLC 2008, Cincinnati, OH 45255 USA

**Keywords:** Traumatic brain injury, Sports injury, Pediatrics

## Abstract

**Background:**

Traumatic brain injuries (TBI) in children result in significant morbidity and mortality. There are many mechanisms, both sport and non-sport related, which cause these injuries. Studies have reported that Emergency Department (ED) visits for pediatric TBI caused by sports are increasing; however, no subsequent study has evaluated the trend in non-sport TBI. The objective of this study was to evaluate ED visits, admissions, and deaths for non-sport TBI compared to those caused by sports.

**Methods:**

A retrospective study of children 5–19 years of age was performed at a pediatric, level 1 trauma center from 2002 to 2012. Subjects with a primary or secondary diagnosis of TBI were identified from the hospital’s trauma registry, and mechanism of injury, disposition, injury severity score, and length of stay were recorded. Frequencies were used to characterize the population, Chi-square analysis was performed to determine differences between groups, and linear trend lines were calculated for sport-related and non-sport TBI by year.

**Results:**

Thirteen thousand two hundred ninty one subjects were seen in the ED between 2002 and 2012 for a TBI; 9527 (72%) were from a non-sport mechanism, and 3764 (28%) were from a sport mechanism. Subjects with a non-sport TBI were more likely to be younger (*p* < 0.001), African American (*p* < 0.001), and have Medicare/Medicaid (*p* < 0.001). Subjects with a non-sport TBI were admitted to the hospital 15% of the time, and subjects with a sport-related TBI were admitted 10% of the time (*p* < 0.001). When evaluating all TBI by mechanism of injury, sport had the lowest injury severity score (mean 4.4) and the shortest length of stay (mean 1.6 days) of any mechanism. There were six deaths reported from non-sport TBI and none from sport-related TBI. ED visits for sport-related TBI increased 92%, and non-sport TBI increased 22% over 10 years. There was a peak in TBI, in both groups, seen in 2009.

**Conclusions:**

ED visits for both sport and non-sport TBI have increased over the past 10 years. TBI from a non-sport mechanism was more likely to result in hospitalization or death. Prevention efforts should be expanded to include all high-risk TBI mechanisms, not just sports.

## Background

Traumatic brain injury (TBI) causes significant morbidity and mortality, resulting in 630,000 Emergency Department (ED) visits, 67,700 hospitalizations, and 6100 deaths annually in children 0–19 years of age (Faul et al. 2010). Nationally, the Centers for Disease Control and Prevention (CDC) have published data demonstrating an increase in nearly 70,000 ED visits for TBI between 2006 and 2010 in this same age group (Faul et al. 2010). In October 2011, the CDC Morbidity and Mortality weekly report published that sport-related TBI is a major culprit for this increase, and subsequent studies have echoed these findings with ED visits from sport-related TBI increasing as much as 92% during the same time period (Hanson et al. 2013).

The cause for the increase in sport-related TBI is not directly clear, but there are several plausible explanations. First, there is greater sports participation in the United States than ever before (National Federation of State High School Associations 2018). Additionally, sport-related TBI has garnered media attention (Metzl 2006; Centers for Disease Control and Prevention 2017; Guskiewicz et al. 2005; Guskiewicz et al. 2007). Media attention increased in 2003 when the CDC launched its Heads Up Campaign aimed to raise awareness and educate the public on prevention and recognition of, and response to TBIs caused by sports. This campaign has had major successes in community settings and physician offices, with large corporate partnerships such as the National Collegiate Athletic Association and the National Football League, and through countless media applications (Stead et al. 2016). Finally, there has been a legislative push for concussion laws that started in the state of Washington in 2009 (Youth Sports-Concussion and head injury guidelines-injured athlete restrictions 2009). By the end of 2013, all 50 states in the United States had a concussion law, often referred to as the “Return to Play” law, that mandates coaches, athletes, and parents be educated on sport-related TBI. This increase in awareness for sport-related injury and more children playing sports likely has contributed to the increase seen in ED visits for this mechanism of TBI.

While the emphasis on sport-related TBI has increased, it seems that attention to the many other mechanisms for TBI – falls, struck by or against an object, motor vehicle crashes (MVCs), motor pedestrian crashes (MPCs), bicycle crashes – has remained stagnant. Most of the current medical literature, educational tools, return to activity and play guidelines, and prevention efforts focus solely on sport-related TBI. Few studies have started to explore the trends in TBI from all mechanisms; however, no studies have specifically explored the relationship between ED visits, hospitalizations, and deaths for non-sport TBI compared to sport-related TBI (Chen et al. 2018; Chen et al. 2017). Our hypothesis was that while sport-related ED visits and hospitalizations have increased over the last 10 years, ED visits and hospitalizations for all mechanisms of non-sport TBI have also increased, but at a lesser rate.

## Methods

### Data source

We performed a retrospective study of children, 5 through 19 years of age, who presented for TBI to a large, pediatric level 1 trauma center, with an annual patient volume of 92,000 (15% admission rate), between January 1, 2002 and December 31, 2011. This age range was chosen because children less than 5 years of age are not consistently involved in organized sports. Patients were identified electronically through the Cincinnati Children’s Hospital Medical Center (CCHMC) Trauma Registry, a hospital-based injury surveillance system that contains information about all patients sustaining injuries. Information in the CCHMC Trauma Registry is obtained by abstracting medical charts of all patients who were treated in the ED and released, were admitted to the hospital, or who died in the ED or hospital as a result of injury (*International Classification of Disease, Ninth Revision* (ICD-9) E-codes 800.00–995.09). Checks are performed monthly by the Trauma Information Coordinator on a random sample of 10% of the charts in the trauma registry to ensure internal consistency and reliability within the database. The hospital’s institutional review board reviewed and approved this study.

### Study population

Subjects were included in this study if they were within the stated age range and had a primary or secondary diagnosis of TBI defined by ICD-9 code (800.0–801.9, 803.0–804.9,850.0–854.1, 950.1–950.3, 995.55, and 959.01). A standard data set was obtained electronically including: medical record number (MRN), age at the time of the injury, gender, race, date of visit/admission, date of injury, disposition (discharged, admission, death), outcome (lived or died), type of insurance coverage, body region injured, mechanism of injury, and ICD-9 code. Admitted patients also had length of stay (LOS) and injury severity score (ISS) recorded.

The definition of sport, both organized and unorganized, included football, basketball, soccer, baseball/softball, skating/blading, swimming, sledding, hockey, gymnastics, and volleyball. The definition of non-sport included all other mechanisms of TBI not included in the definition of sport – including, but not limited to falls, abuse, struck by or against mechanism, bicycle crashes, MVCs, and MPCs. MVC refers to any person injured as a driver or passenger of a vehicle, and MPC refers to any person struck by a vehicle while operating as a pedestrian outside the vehicle.

### Statistical analysis

The data for all subjects meeting inclusion and exclusion criteria were entered into Statistical Package for the Social Sciences (Release 19.0.0, 2010, IBM SPSS Statistics, IBM Corporation) database. Frequencies were calculated to define the study population. Chi square was used to determine differences between those children with sport-related TBI and those with non-sport TBI. A *p*-value < 0.05 was considered statistically significant. Linear trend lines were calculated for sport-related and non-sport TBI by year.

## Results

**Fig. 1:**
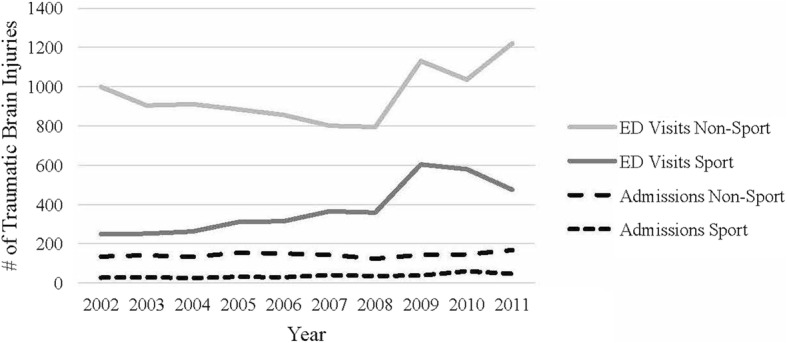
Number of emergency department visits and admissions for non-sport and sport-related traumatic brain injury by year

In our ED, 14,166 patients aged 5–19 years were seen between 2002 and 2012 with a primary or secondary diagnosis of TBI. There were 726 patients who were excluded because no mechanism of injury was recorded. One hundred and 49 patients presented to the ED more than once within 7 days of their injury and were excluded. The final study cohort included 13,291 patients. This represented 2.6% of all patients seen during the same time period (*n* = 510,198) in the CCHMC ED. Non-sport mechanisms resulted in 9528 (71.7%) TBIs, and 3763 (28.3%) were a result of a sports mechanism. Table 1 shows demographics of patients sustaining non-sport TBI versus those sustaining sport-related TBI. The children with non-sport TBI were younger (mean age 11.0, SD 3.9) than those with sport-related TBI (mean age 13.2, SD 3.2). Also, children with non-sport TBI were more likely to be female (73% vs 63%), were more likely to be African American (27% vs 16%), and had a higher percentage of Medicare/Medicaid (28% vs 11%). In addition, more patients with non-sport TBI died and were admitted to the hospital. When only looking at a primary diagnosis of TBI, excluding the secondary diagnosis, there was no difference in deaths, but there was a higher rate of hospitalization for primary TBI diagnosis (13.9%) than secondary diagnosis (9.9%). Visits to the ED between 2002 and 2012 for sport-related TBI increased 92% and non-sport TBI increased 22% while admissions for both mechanisms remained stable (Fig.1).

**Table 1:** Demographics for children with sport versus non-sport traumatic brain injury

	Sport (*n* = 3763)	Non-Sport (*n* = 9528)	p-value sport versus non-sport
Age			< 0.001
5–9 years	644 (17.1%)	4335 (45.5%)	
10–14 years	1832 (48.7%)	3276 (34.4%)	
15–18 years	1287 (34.2%)	1917 (20.1%)	
Gender			< 0.001
Male	2746 (73.0%)	5951 (62.5%)	
Race			< 0.001
African American	596 (15.8%)	2612 (27.4%)	
Caucasian	2934 (78.0%)	6282 (65.9%)	
Other	226 (6.0%)	620 (6.5%)	
Unknown	7 (0.2%)	14 (0.1%)	
Insurance			< 0.001
Medicare/Medicaid	407 (10.8%)	2672 (28.0%)	
Private	3183 (84.6%)	6102 (64.0%)	
Self-Pay	68 (1.8%)	198 (2.1%)	
Other/Unknown	105 (2.8%)	556 (5.9%)	
Emergency Department Disposition			
Admission	361 (9.6%)	1409 (14.8%)	< 0.001
Death	0 (0.0%)	29 (0.3%)	
Home	3402 (90.4%)	8090 (84.9%)	
Hospital Disposition	(*n* = 361)	(*n* = 1438)	0.002
Death	0 (0.0%)	29 (2.0%)	
Home	361 (100%)	1409 (98.0%)

**Fig. 2:**
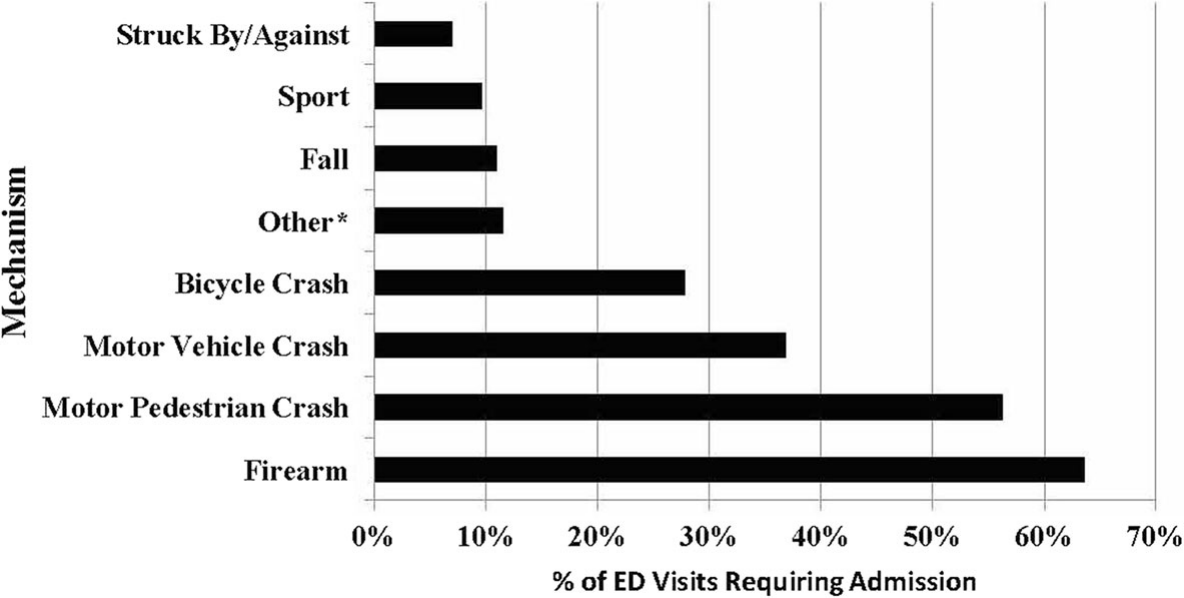
Percentage of emergency departments visits requiring admission by mechanism. *Other includes drowning/submersion, burn, abuse, or penetrating injury

**Fig. 3:**
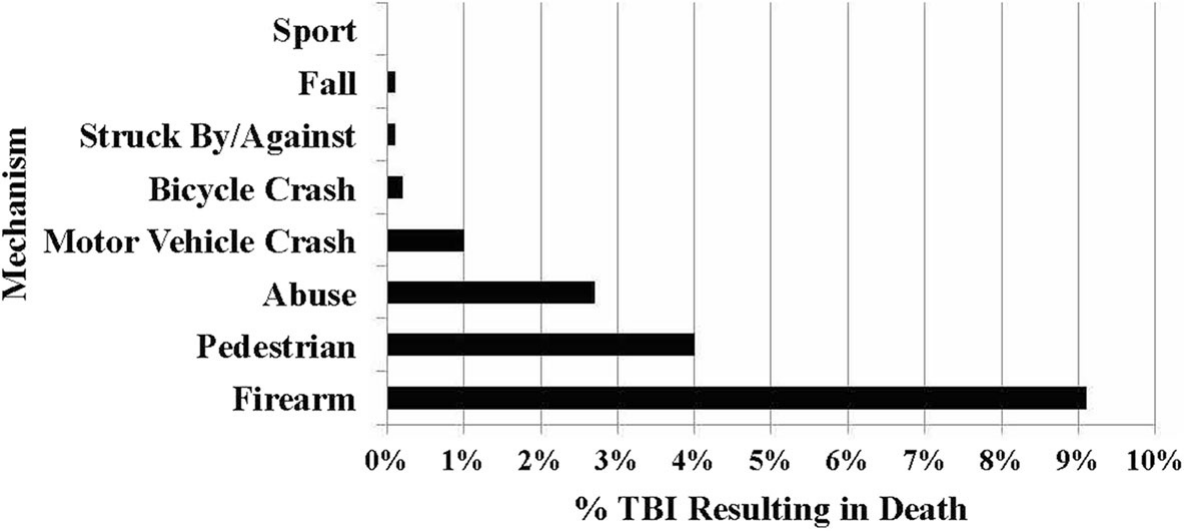
Percentage of traumatic brain injury resulting in death by mechanism

Of all mechanisms causing TBI, firearm injuries (64%), MPC (56%), MVC (37%), and bicycle crash (28%) were the most likely to require admission (Fig. 2). Firearm injuries (9%), MPCs (4.0%), abuse (2.7%), and MVC (1.0%) were the most likely TBI mechanisms to result in death (Fig. 3). There were 29 deaths from non-sport TBI and zero from sport-related TBI. Of the patients that were admitted to the hospital, those with TBI caused by a sport mechanism had the lowest ISS (median = 4.0) and the shortest length of stay (mean = 1.6 days) of any mechanism (Table 2).

**Table 2:** Injury severity score and length of stay for admitted patients by mechanism

Mechanism	Injury Severity Score on Admission (median)	Length of Stay (mean, days)
Sport	4	1.6
Unknown	4	1.4
Struck By/Against	6	2.6
Fall	9	2.0
Bicycle Crash	9	2.2
Burn/Drowning/Penetrating	9	8.9
Motor Vehicle Crash	10	3.1
Motor Pedestrian Crash	10	4.1
Firearm	10	9.9
Abuse	31	3.5

## Discussion

Pediatric TBIs cause significant morbidity and mortality (Harmon et al. 2013). Recent research has demonstrated that TBI resulting from sports is increasing and many of our efforts in research, prevention, and education on TBI has focused specifically on this mechanism (Hanson et al. 2013; Centers for Disease Control and Prevention 2011; Giza et al. 2013; McCrory et al. 2009) This study evaluated trends in non-sport mechanisms. We showed that there was an increase in ED visits for non-sport TBI, and these mechanisms result in greater numbers of admissions, longer LOS, higher ISS, and more deaths, when compare to sport-related TBI.

The demographic statistics of our populations were consistent with the literature. It has been established that more older children play sports than younger children (Yang et al. 2008). Additionally, while prior work has shown that there is greater ED utilization for injuries in children of lower socioeconomic status, this paradigm has not held true for sport-related injuries (Simon et al. 2004; Faelker et al. 2000). Sport injuries have been linked to higher socioeconomic status, and we see this reflected in the greater proportion of privately insured patients with sport-injuries (Ni et al. 2002). Prior research has also shown disparity in the participation of racial minorities in organized sports, in part due to social factors, physical education availability, and community infrastructure (Richmond et al. 2006; Johnston et al. 2007; Sheu and Chen 2016).

In our population, ED visits for TBI from both sport and non-sport mechanism have increased over the last 10 years. We saw a 92% increase in sport-related TBI and a 22% increase in non-sport TBI, with the greatest increase visible in 2009. It is not clear from our data what may have triggered this sharp increase. We know from our data, however, that admissions for TBI from both mechanisms remained stable, which indicates the increase in ED visits was largely from minor TBI. Our hypothesis is this may have been a result of increased attention towards the morbidity and mortality related to TBI, particularly with the passing of the Zachary Lystedt law in 2009 (Youth Sports-Concussion and head injury guidelines-injured athlete restrictions 2009). It should be noted that Ohio concussion legislation went into effect in 2013. The states bordering the region, Kentucky and Indiana, enacted concussion legislation in 2011 and 2012, respectively. The cause of this trend is likely multifactorial and would need to be explored in detail with another study.

ED visits for sports-related TBI are increasing at a greater rate than ED visits for non-sport TBI. There are a few plausible explanations for this difference. Sport-related injuries may now be classified differently (i.e., instead of struck-by/against mechanism). Additionally, the increased recognition of the danger of sport-related TBI may have resulted in more children presenting to the ED with these injuries (Metzl 2006; Centers for Disease Control and Prevention 2017). It should also be noted that non-sport injuries are under-represented as children < 5 years were not included in this study, and these children are more likely to be seen for TBI from mechanisms other than sports. The rate of change for non-sport TBI may be higher than reported in this study.

This study showed that at our institution non-sport TBI are responsible for the most severe TBIs in terms of injury severity, LOS, and death. Those TBIs that are secondary to high velocity mechanisms are still responsible for the injuries that have the greatest morbidity and mortality. This is consistent with CDC data from 2006 to 2010 that showed falls to be the leading cause of TBI-related hospitalization in children 0–14 years of age and MVCs for children 15–18 years of age (Taylor et al. 2017). It should be emphasized that 4% of children, in our study, who sustained a TBI from a MPC died. Excluding children < 5 years of age in our study potentially blunts the true morbidity and mortality associated with non-sport TBI.

Given the more severe injury caused by non-sport-related TBI, legislation on prevention of these mechanisms and education with return to play/school guidelines should be robust. For instance, MVCs have legislation such as car seat and seatbelt laws to help prevent serious injury. Other non-sport TBI mechanisms such as bicycle crashes, falls, and firearm injuries have tools available such as helmets, window-locks, and gun safes and work being done by specific prevention groups. An argument can be made, however, that since these mechanisms are still responsible for the most severe injuries in children, a renewed emphasis on education and prevention is warranted. The CDC released a guideline in November 2017 for the diagnosis and management of mild traumatic brain injury, focused on concussions, in children that will hopefully continue to move the discussion forward in terms of simplifying terminology and recommending treatment (Lumba-Brown et al. 2018).

There are limitations to this study. First, this was a retrospective study performed at a single, Midwest pediatric level 1 trauma center. The findings may not be generalizable to other populations. Secondly, coding errors are possible. Those who maintain the trauma registry review the medical chart carefully to limit this. Using the ISS variable from the trauma registry, as opposed to using a calculated Abbreviated Injury Severity (AIS) score, which is a better measure of TBI severity, was another limitation. In addition, we looked at volumes of injuries and not rates so, for example, sports injuries may have increased because the volume of children participating in sports increased. Lastly, we looked at only those subjects who had TBI listed as a primary or secondary diagnosis. Thus, we may have missed subjects with multi-trauma; however, our aim was to limit the inclusion of TBI that was inconsequential to the patient.

## Conclusions

ED visits for pediatric sport-related TBI account for the majority of TBIs seen in children 10–19 years of age; however, ED visits for both sport-related and non-sport TBI are increasing. Admissions and deaths from TBI are more common in non-sport injuries. Education and prevention efforts should focus on the other high-risk mechanisms, in addition to sport-related TBI.

## Abbreviations

AIS: Abbreviated injury severity
CCHMC: Cincinnati Children’s Hospital Medical Center
CDC: Centers for Disease Control and Prevention
ED: Emergency Department
ICD-9: International Classification of Diseases Ninth Revision
ISS: Injury severity score
LOS: Length of stay
MPC: Motor pedestrian crash
MRN: Medical record number
MVC: Motor vehicle crash
TBI: traumatic brain injury

## References

[CR1] Centers for Disease Control and Prevention (2011). Nonfatal traumatic brain injuries related to sports and recreation activities among persons aged <19 years - United States, 2001–2009. MMWR.

[CR2] Centers for Disease Control and Prevention. HEADS Up. National Center for Injury Prevention and Control, Division of Unintentional Injury Prevention. http://www.cdc.gov/headsup/index.html. Published June 2017. Accessed Nov 2018.

[CR3] Chen Cheng, Peng Jin, Sribnick Eric, Zhu Motao, Xiang Henry (2018). Trend of Age-Adjusted Rates of Pediatric Traumatic Brain Injury in U.S. Emergency Departments from 2006 to 2013. International Journal of Environmental Research and Public Health.

[CR4] Chen Cheng, Shi Junxin, Stanley Rachel, Sribnick Eric, Groner Jonathan, Xiang Henry (2017). U.S. Trends of ED Visits for Pediatric Traumatic Brain Injuries: Implications for Clinical Trials. International Journal of Environmental Research and Public Health.

[CR5] Faelker T, Pickett W, Brison RJ (2000). Socioeconomic differences in childhood injury: a population based epidemiologic study in Ontario. Inj Prev.

[CR6] Faul M, Xu L, Wald MM, Coronado VG (2010). Traumatic brain injury in the United States: emergency department visits, hospitalizations and deaths 2002–2006.

[CR7] Giza CC, Kutcher JS, Ashwal S (2013). Summary of evidence-based guideline update: evaluation and management of concussion in sports: report of the guideline development Subcommittee of the American Academy of neurology. Neurology.

[CR8] Guskiewicz KM, Marshall SW, Bailes J (2005). Association between recurrent concussion and late-life cognitive impairment in retired professional football players. Neurosurgery.

[CR9] Guskiewicz KM, Marshall SW, Bailes J (2007). Recurrent concussion and risk of depression in retired professional football players. Med Sci Sports Exerc.

[CR10] Hanson HR, Pomerantz WJ, Gittelman M. ED utilization trends in sports-related traumatic brain injury. Pediatrics. 2013;132(4):e859–64. 10.1542/peds.2013-1704 PMC4530300 24081999

[CR11] Harmon KG, Drezner J, Gammons M (2013). American medical Society for Sports Medicine position statement: concussion in sport. Clin J Sport Med.

[CR12] Johnston LD, Delva J, O'Malley PM (2007). Sports participation and physical education in American secondary schools: current levels and racial/ethnic and socioeconomic disparities. Am J Prev Med.

[CR13] Lumba-Brown A, Yeates KO, Sarmiento K (2018). Centers for Disease Control and Prevention Guideline on the Diagnosis and Management of Mild Traumatic Brain Injury Among Children. JAMA Pediatr.

[CR14] McCrory P, Meeuwisse W, Johnston K (2009). Consensus statement on Concussion in sport--the 3rd international conference on Concussion in sport held in Zurich, November 2008. J Science Med. Sport.

[CR15] Metzl JD (2006). Concussion in the young athlete. Pediatrics.

[CR16] National Federation of State High School Associations. High school sports participation increases for 29^th^ consecutive year. National Federation of State High School Associations. http://www.nfhs.org/articles/high-school-sports-participation-increases-for-29th-consecutive-year?ArtId=205458. Accessed Nov 2018. Published Sept 2018.

[CR17] Ni H, Barnes P, Hardy AM (2002). Recreational injury and its relation to socioeconomic status among school aged children in the US. Inj Prev.

[CR18] Richmond TK, Hayward RA, Gahagan S, Field AE, Heisler M (2006). Can school income and racial/ethnic composition explain the racial/ethnic disparity in adolescent physical activity participation?. Pediatrics.

[CR19] Sheu Y, Chen LH. Hedegaard H. Sports- and Recreation-related Injury Episodes in the United States 2011-2014. Natl Health Stat Rep. 2016;99:1–12. 27906643

[CR20] Simon TD, Bublitz C, Hambidge SJ (2004). External causes of pediatric injury-related emergency department visits in the United States. Acad Emerg Med.

[CR21] Stead TS, Rastogi V, Hedna VS, Ganti L (2016). Awareness of the CDC "Heads up!" to youth Sports campaign among pediatric Sports coaches: a pilot survey study. Cureus.

[CR22] Taylor CA, Bell JM, Breiding MJ, Xu L (2017). Traumatic brain injury-related emergency department visits, hospitalizations, and deaths - United States, 2007 and 2013. MMWR Surveill Summ.

[CR23] Yang J, Phillips G, Xiang H, Allareddy V, Heiden E, Peek-Asa C (2008). Hospitalisations for sport-related concussions in US children aged 5 to 18 years during 2000-2004. British Journal of Sports Medicine.

[CR24] Youth Sports-Concussion and head injury guidelines-injured athlete restrictions. State of Washington, 61^st^ Legislature. 2009 Regular Session. http://apps.leg.wa.gov/rcw/default.aspx?cite=28a.600.190. Accessed Nov 2018.

